# Detection of Pneumococcal Carriage in Asymptomatic Healthcare Workers

**DOI:** 10.1093/ofid/ofaf008

**Published:** 2025-01-15

**Authors:** Pari Waghela, Raechel Davis, Melissa Campbell, Rupak Datta, Maikel S Hislop, Noel J Vega, Loren Wurst, Devyn Yolda-Carr, Luke Couch, Michael Hernandez, Lindsay R Grant, Ronika Alexander-Parrish, Adriano Arguedas, Bradford D Gessner, Richard A Martinello, Daniel M Weinberger, Anne L Wyllie

**Affiliations:** Department of Epidemiology of Microbial Diseases, Yale School of Public Health, New Haven, Connecticut, USA; Department of Epidemiology of Microbial Diseases, Yale School of Public Health, New Haven, Connecticut, USA; Division of Pediatric Infectious Diseases, Department of Pediatrics, Duke University School of Medicine, Duke University, Durham, North Carolina, USA; Department of Internal Medicine, Section of Infectious Diseases, Yale School of Medicine, New Haven, Connecticut, USA; Department of Epidemiology of Microbial Diseases, Yale School of Public Health, New Haven, Connecticut, USA; Department of Epidemiology of Microbial Diseases, Yale School of Public Health, New Haven, Connecticut, USA; Department of Epidemiology of Microbial Diseases, Yale School of Public Health, New Haven, Connecticut, USA; Department of Epidemiology of Microbial Diseases, Yale School of Public Health, New Haven, Connecticut, USA; Department of Epidemiology of Microbial Diseases, Yale School of Public Health, New Haven, Connecticut, USA; Department of Epidemiology of Microbial Diseases, Yale School of Public Health, New Haven, Connecticut, USA; Global Respiratory Vaccines, Pfizer, Inc., Collegeville, Pennsylvania, USA; Global Respiratory Vaccines, Pfizer, Inc., Collegeville, Pennsylvania, USA; Global Respiratory Vaccines, Pfizer, Inc., Collegeville, Pennsylvania, USA; Global Respiratory Vaccines, Pfizer, Inc., Collegeville, Pennsylvania, USA; Department of Internal Medicine, Section of Infectious Diseases, Yale School of Medicine, New Haven, Connecticut, USA; Department of Epidemiology of Microbial Diseases, Yale School of Public Health, New Haven, Connecticut, USA; Department of Epidemiology of Microbial Diseases, Yale School of Public Health, New Haven, Connecticut, USA

**Keywords:** carriage, pandemic, pneumococcus, saliva, surveillance

## Abstract

**Background:**

Healthcare workers are at increased risk of exposure to respiratory pathogens including *Streptococcus pneumoniae* (pneumococcus). While little asymptomatic carriage has been reported in young-to-middle-aged adults, this may be due to nonsensitive diagnostic methods. The aim of the current study was to investigate the rates of pneumococcal carriage in a large cohort of healthcare workers, using saliva as a respiratory specimen.

**Methods:**

We evaluated pneumococcal carriage in convenience samples of saliva, self-collected from asymptomatic healthcare workers (Connecticut, USA) who were testing for severe acute respiratory syndrome coronavirus 2 (SARS-CoV-2) from 30 March to 11 June 2020. DNA extracted from the culture-enriched saliva was later tested using quantitative polymerase chain reaction for *piaB*, *lytA*, and serotype. Saliva samples were considered positive for pneumococcus when the *piaB* cycle threshold value was <40.

**Results:**

Study participants were 22–74 years old (mean age, 38.5 years), 75% female, 75% white, and with occupations including registered nurses (48%), medical doctors (23%), and patient care assistants (5%). Overall, 138 of 1241 samples (11%) from 86 of 392 individuals (21%) tested *piaB* positive at some point during the 4-month study period, with 28 (33%) colonized individuals positive at multiple time points. Carriers reflected the overall study population. No significant demographic characteristics were associated with detection of pneumococcus. Colonized individuals primarily carried serotypes 19F (25.6%) and 3 (12.8%).

**Conclusions:**

During a period of mandatory masking, we identified a cumulative pneumococcal carriage prevalence of 21% among healthcare workers. This study highlights that healthcare workers may act as unrecognized reservoirs of pneumococcus in the population. Despite long-standing pediatric immunization programs, vaccine-targeted serotypes continue to be prevalent among the adult population.

A common commensal inhabiting the upper respiratory tract, *Streptococcus pneumoniae* (pneumococcus) is also a leading cause of respiratory infections, such as otitis media and sinusitis, and life-threatening, invasive pneumococcal diseases, including pneumonia, bacteremia, and meningitis. Children are considered the reservoir of pneumococcus in the population, with carriage rates typically reported to decline with increasing age, with little asymptomatic carriage reported in young-to-middle-aged adults [1]. Prior studies have demonstrated that contact with children is associated with higher pneumococcal carriage prevalence in adults [1, 2], including healthcare workers working in the pediatric setting [3]. Since healthcare workers are often exposed to the respiratory secretions of sick individuals, it is not just those working in pediatrics who are potentially at an increased risk of exposure to respiratory pathogens including pneumococcus.

Pneumococcal carriage has seldom been investigated and reported among healthcare workers, despite their potentially elevated risk. Elucidating the prevalence of pneumococcal carriage in this population is important not only for informing occupational risks but also for understanding potential transmission dynamics within healthcare settings. Healthcare workers, who are frequent carriers of pneumococci, may pose an increased risk of transmission to vulnerable populations, such as individuals who are elderly, immunocompromised, or have chronic illnesses. Understanding this risk can inform the development of strategies to minimize spread among both healthcare workers and patients. Therefore, in the current study, we evaluated pneumococcal carriage rates and serotypes in a cohort of healthcare workers at Yale New Haven Hospital.

## METHODS

### Ethics

Medical staff at Yale New Haven Hospital were eligible to participate in the study voluntarily. All study participants were enrolled and sampled in accordance with Yale University Human Investigation Committee–approved protocol 2000027690. Demographics, clinical data, and samples were collected after the study participant acknowledged that they understood the study protocol and provided informed consent. All participant information and samples were collected in association with study identifiers that were not individually identifiable.

### Participant Enrollment

Between 30 March and 11 June 2020, healthcare workers without fever or respiratory symptoms working within Yale New Haven Hospital, on inpatient coronavirus disease 2019 (COVID-19) or non–COVID-19 units, emergency or ambulatory care departments, or with other occupational exposure to patients, were invited to enroll in the Yale IMPACT biorepository study [4]. This study was designed to actively monitor asymptomatic healthcare workers for severe acute respiratory syndrome coronavirus 2 (SARS-CoV-2) infection. Individuals were not included in the study if they were non–English speaking or <18 years of age.

### Sample Collection and Processing

Healthcare workers were invited to self-collect saliva every 3 days for up to 84 days, or until testing positive for SARS-CoV-2. Samples were stored at +4°C until transport to the research laboratory, where they were tested for SARS-CoV-2 [4]. The remnant sample volume was stored at −80°C within 12 hours. Starting in July 2021, saliva samples were thawed on ice, vortexed to resuspend, and 100 µL was plated on TSAII plates with 5% sheep's blood and 10% gentamicin, as described elsewhere [2]. Following overnight incubation, bacterial growth was harvested into brain-heart infusion medium supplemented with 10% glycerol. These culture-enriched saliva samples were then stored at −80˚C until further analysis. We previously validated this method to test the viability of pneumococcus when stored and tested through this approach [2, 5].

### Pneumococcal Detection

Culture-enriched samples were thawed on ice, then DNA was extracted from 200 µL of each sample using the MagMAX Viral/Pathogen Nucleic Acid Isolation Kit on the KingFisher Apex with a modified protocol [2]. Each DNA template was tested using quantitative polymerase chain reaction (qPCR) targeting pneumococcal-specific genes p*iaB* (encoding the iron acquisition ABC transporter lipoprotein PiaB) and *lytA* (encoding the major autolysin LytA), as described elsewhere [2]. Samples were considered positive with *piaB* cycle threshold (Ct) values <40.

### Strain Isolation

Saliva samples that tested positive for *piaB* with a Ct <28 were revisited by culture [2] or magnetic bead-based separation [6] in an attempt to isolate pure pneumococcus. Isolates were serotyped by latex agglutination (Statens Serum Institut) [7].

### Serotype Determination

DNA extracts that tested positive for *piaB* were pooled by 4, and those that tested negative were pooled by 10. Where possible, samples from the same individual were pooled together. Each pool was tested in 8 multiplexed serotyping assays [8] targeting a total of 39 serotypes (see Supplementary Information) [9–12]. From each pool, 8 µL of DNA was tested in a total reaction volume of 25 µL. All samples from any pool generating a serotype-specific Ct value <40 were tested individually in that serotyping assay. The primary serotype of an individual sample was determined based on concordance between the *piaB* Ct value and the serotype-specific Ct value. Assay reliability was determined based on the lack of serotype-specific signal in *piaB*-negative pools and/or the lack of serotype-specific signal that was >4 Ct lower than the *piaB* Ct value of positive samples.

### Data Analysis

Differences in characteristics between pneumococcal carriage groups were tested using the Kruskal-Wallis rank sum test (continuous variable) or Fisher exact test (categorical variable). Estimates were considered statistically significant at *P* < .05. Graphical visualizations and statistical analyses were performed with RStudio (version 2022.07.2-576), using R software (version 4.1.3) [13]. Data preprocessing and visualization was performed using tidyverse [14] and table1 [15] R packages.

## RESULTS

### Study Population

A total of 525 healthcare workers provided informed consent for inclusion in the Yale IMPACT biorepository study [4]. Of those, 16 (3%) tested positive for SARS-CoV-2 [16] and were excluded from this study due to limited power to describe coinfection. Saliva samples from 483 of 509 study participants (95%) were available for further analyses. From this cohort, we selected 392 of 483 (81%) to test for pneumococcus. Study participants were selected to represent the demographic makeup of the total study population, and on availability of sufficient sample volume for testing. Full demographic characteristics of the study population are shown in Table 1. Vaccination status, medical history, and information on living situations were not available.

**Table 1.** Characteristics of the Study Population, Overall and by Detection of Pneumococcal Carriage

Characteristic	Study Participants, No. (%)^a^		
	Negative (n = 306)	Positive (n = 86)	Overall (n = 392)
Age			
Mean (SD), y	38.4 (11.8)	38.7 (10.7)	38.5 (11.5)
Median (range), y	35.0 (23.0– 74.0)	37.0 (22.0– 67.0)	35.0 (22.0–74.0)
Missing	10 (3.3)	2 (2.3)	12 (3.1)
Sex			
Female	231 (75.5)	64 (74.4)	295 (75.3)
Male	67 (21.9)	20 (23.3)	87 (22.2)
Missing	8 (2.6)	2 (2.3)	10 (2.6)
Ethnicity			
White	230 (75.2)	63 (73.3)	293 (74.7)
Hispanic or Latino	27 (8.8)	6 (7.0)	33 (8.4)
Asian	20 (6.5)	12 (14.0)	32 (8.2)
Black or African American	14 (4.6)	2 (2.3)	16 (4.1)
Other	5 (1.6)	1 (1.2)	6 (1.5)
Missing	10 (3.3)	2 (2.3)	12 (3.1)
Occupation			
Registered nurse	141 (46.1)	48 (55.8)	189 (48.2)
Medical doctor	72 (23.5)	20 (23.3)	92 (23.5)
Patient care assistant	18 (5.9)	4 (4.7)	22 (5.6)
Physician assistant	8 (2.6)	7 (8.1)	15 (3.8)
Charge nurse	9 (2.9)	0 (0)	9 (2.3)
Nurse practitioner	8 (2.6)	0 (0)	8 (2.0)
Radiology technologist	3 (1.0)	2 (2.3)	5 (1.3)
Nurse manager	4 (1.3)	0 (0)	4 (1.0)
Respiratory therapist	4 (1.3)	0 (0)	4 (1.0)
Other	31 (10.1)	3 (3.5)	34 (8.7)
Missing	8 (2.6)	2 (2.3)	10 (2.6)

^a^Data represent no. (%) of study participants unless otherwise specified.

### Pneumococcal Carriage

**Figure 1.**
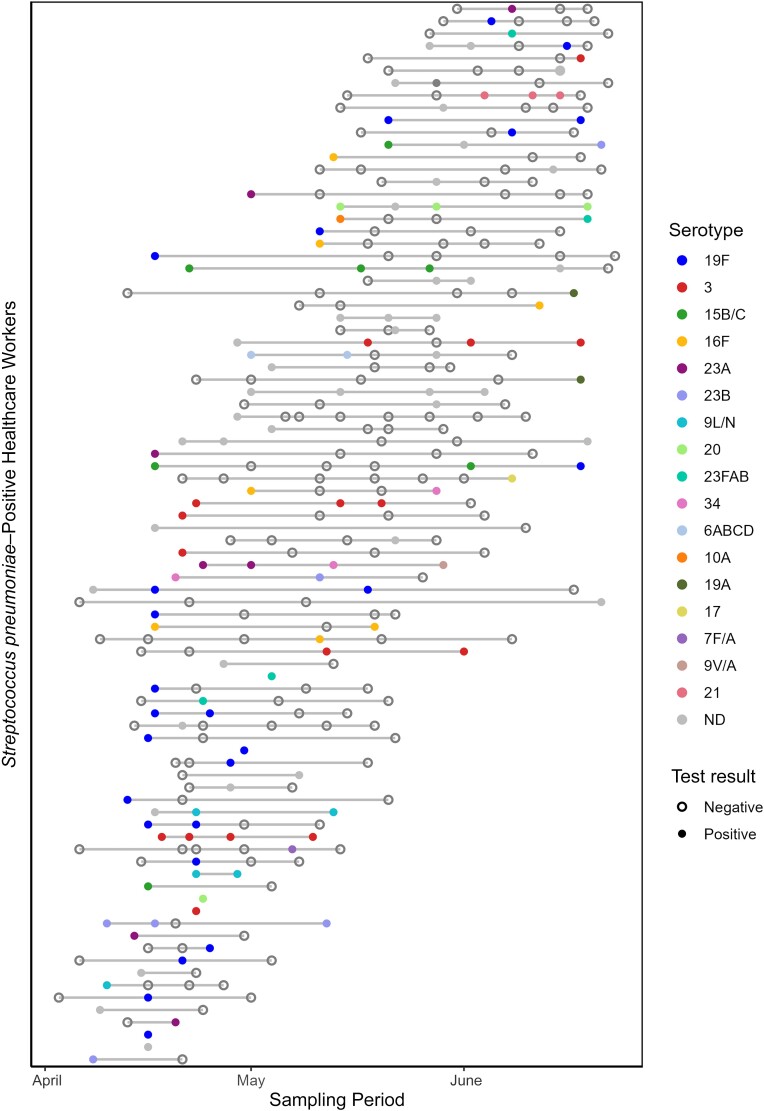
Detection of pneumococcus and pneumococcal serotype in self-collected saliva samples from asymptomatic healthcare workers, April–July 2020. All samples from any individual who tested positive for pneumococcus (*piaB*) with quantitative polymerase chain reaction (qPCR) at least once during the study period are shown. Consecutive samples from the same individual are joined by a solid gray line. Solid circles denote samples qPCR positive for *piaB,* colored according to the primary serotype result. Secondary serotypes detected are not shown. Gray solid circles represent samples for which a serotype could not be determined (ND) using the available qPCR assays, and open circles denote samples qPCR negative for *piaB.* The serotypes in the legend are ordered by decreasing prevalence among the study population.

Overall, 1241 samples from 392 individuals were tested for pneumococcus (range, 1–10 samples per individual; mean, 3.17 per individual). Of those, 138 samples (11%) tested qPCR positive for *piaB*, with 86 of 392 individuals (21%) testing positive on ≥1 sampling moment during the 4-month study period (Figure 1); 28 of 86 carriers (33%) tested positive more than once. The average duration between positive sampling moments was 15.2 days, while the shortest duration between positive samples was 4 days and the longest 53 days.

Positive individuals reflected the overall study population (Table 1) and were 22–67 years old (mean age, 38.7 years), 74% female, and 73% white, with occupations including registered nurse (55%), medical doctor (23%), physician assistant (8%), and patient care assistant (4%); demographics by occupation are available in Supplementary Table 1.

Positivity for pneumococcal carriage was defined as detection of *piaB* [2] due to known nonspecificity of the *lytA* qPCR assay leading to cross-detection of other streptococci [17]. In line with this, 287 of 1231 samples (23%) from 145 of 392 individuals (37%) tested positive for *lytA* (Ct <40) but negative for *piaB* (Ct >40). In contrast, 18 samples (1.5%) from 15 individuals (4%) were positive for *piaB* but negative for *lytA*. These *piaB*-positive/*lytA-*negative results were confirmed through retesting. In addition, 8 (44%) of the 18 *piaB-*positive only samples from 6 (40%) of 15 individuals also returned a serotype-specific Ct value that aligned with that obtained for *piaB*.

### Serotype Determination

**Figure 2.**
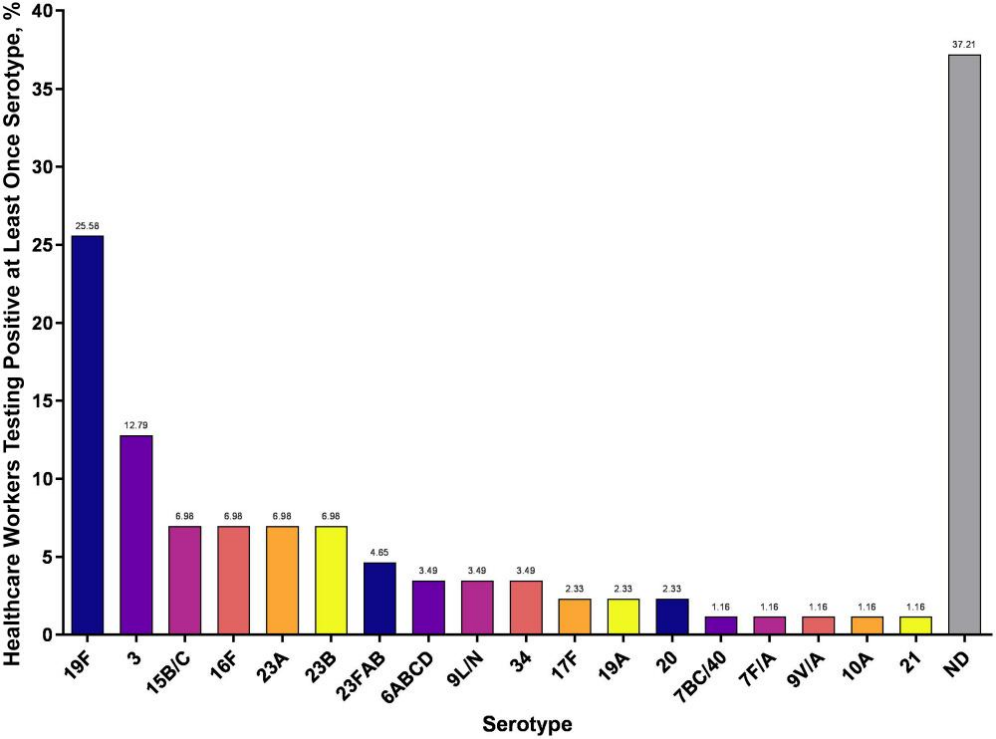
Percentage of healthcare workers who tested positive at least once for the pneumococcal serotypes detected in the study population. The percentage of positive individuals is also shown at the top of each bar. Abbreviation: ND, not determined (ie, a serotype could not be determined using the available assays).

Of the 27 single-plex serotyping assays within the 8 multiplexed assays, we determined 20 of the assays to be reliable (2 [12], 3 [12], 6ABCD [18], 7BC/40 [9], 7F/A [12], 9L/N [12], 9 V/A [9], 10A [12], 11ADE [12], 14 [18], 15B/C [12], 16F [18], 17F [12], 19A [10], 19F [12], 21 [12], 23A [12], 23B [12], 23FAB [9], and 34 [12]), representing 31 of 106 pneumococcal serotypes. From these, we detected 108 serotype-specific signals, 96 that we considered as the primary serotype (within 4 Ct of *piaB*; average Ct value, 33.07; range, 17.70–39.89) and 12 that we considered as secondary serotypes (>4 Ct weaker than *piaB*; average Ct, 35.13; range, 24.88–39.33). From this panel, we were unable to determine a primary serotype in 39 (28.3%) of 138 *piaB*-positive samples from 30 (34.9%) of 86 pneumococcus-positive individuals. Overall, 25.6% of pneumococcus-positive individuals (and 19.6% of samples) tested positive for serotype 19F at least once over the study period, and 12.8% of positive individuals (and 14.5% of samples) tested positive at least once for serotype 3. Primary serotypes detected are presented in Figure 1, and all serotypes detected within all pneumococcus-positive individuals are presented in Figure 2; 7BC/40 was only detected as a secondary serotype, generating signal in 2 samples from a single individual 6 days apart.

Pneumococcal isolates were recovered from 4 samples. Three isolates from 2 individuals were confirmed to be serotype 3, and 1 isolate from another individual was confirmed to be serogroup 23 by means of latex agglutination. For all samples, results from latex agglutination were consistent with serotype detection by qPCR.

## DISCUSSION

Most previous adult carriage studies have reported low pneumococcal prevalence [19, 20] based on nasopharyngeal swab samples tested using conventional culture methods. We and others have demonstrated that testing oral sample samples with qPCR significantly increases the sensitivity of carriage detection in adults [21–23]. Applying this approach to improve pneumococcal carriage detection among young-to-middle-aged adults also revealed that pneumococcal colonization is more prevalent among parents of young children than among adults without children [1]. A similar finding was reported among healthcare workers of a pediatric hospital [3], likely also due to the high risk of transmission from children. In this study we applied our saliva-based qPCR methods to investigate carriage of pneumococcus among a broader population of healthcare workers. Our findings highlight that the higher prevalence of pneumococcal carriage is not limited to those working with children in pediatric departments or hospitals [3] but is likely more widespread across the entire hospital setting. The source of carriage acquisition, whether from the patient population or outside contacts, such as children, is unclear.

The point prevalence of 11% and period prevalence of 21% for pneumococcal carriage in this study population were observed during the early COVID-19 pandemic when strict nonpharmaceutical interventions for mitigating SARS-CoV-2 transmission were in place and strictly adhered to and when invasive pneumococcal diseases had declined globally. While we do not have data on the carriage prevalence among this population of healthcare workers before the COVID-19 pandemic, a number of studies, including our own study of older adults living in the same New Haven community [2], have demonstrated that pneumococcal carriage persisted in both children [24, 25] and adults [2, 8] during this time, despite the nonpharmaceutical interventions in place. It is therefore possible that, in the typical absence of these transmission mitigation measures and with a return to the typical annual circulation of respiratory pathogens [26] and their associated respiratory infections, a higher rate of pneumococcal transmission may occur in this population, and higher rates of colonization may even occur.

While the recommended sample type for the detection of pneumococcus in adults is the nasopharyngeal swab sample, with the addition of an oropharyngeal swab when possible [27], saliva samples have been effective for enhancing detection [1, 21, 28]. Combined with qPCR, this is one of the most sensitive approaches for detecting pneumococcus [29]. Despite its higher sensitivity, our group has demonstrated nonspecificity of the *lytA* assay when applied to saliva [17], due to the cross-detection of gene homologues present in nonpneumococcus streptococci. In a study of pediatric healthcare workers by Steurer et al [3], the authors recognized this as a potential limitation of their study, in which oropharyngeal swab samples were tested using qPCR targeting *lytA* only. While the specificity of their results would need to be confirmed through testing for additional gene targets [30], the prevalence detected in their cohort of pediatric healthcare workers was not too different from our own observations here.

This risk of cross-detection also applies to molecular methods of serotype detection [31], with nonpneumococcal streptococci also harboring capsular biosynthesis genes that can be identical to pneumococcal genes, at least in part. We applied a strict assessment of the specificity of each qPCR assay through the testing of negative samples and critically evaluation of the Ct values generated by assays of higher specificity to determine serotyping reliability. However, we do recognize that a limitation of our approach is the difficulty in isolating individual pneumococcal colonies from microbially dense saliva samples to confirm the results of molecular detection and to identify serotypes for samples that tested positive in qPCR for *piaB* but negative in our limited panel of serotyping qPCR assays. Despite this, we observed a high prevalence of serotype 19F, detected at least once in 25.6% of carriers, and of serotype 3, detected at least once in 12.8% of carriers, with the observed rate of positivity for 19F higher than reports from other carriage studies in recent years [32]. While both of these serotypes are targeted by 13-valent pneumococcal conjugate vaccine (the only conjugate vaccine recommended in the United States through the period of the study), these serotypes have persisted as causes of invasive pneumococcal diseases [33, 34].

The prevalence and dynamics of pneumococcal carriage in healthcare settings should be an important consideration for community transmission, particularly to at-risk individuals. Our study demonstrated a notable prevalence of pneumococcal carriage among healthcare workers, extending beyond those working in pediatric settings, and despite extensive use of nonpharmaceutical interventions during the study period. Together with the high frequency of detection of serotypes targeted by long-standing PCV13 immunization programs, this work contributes to the growing body of literature indicating that pneumococcal carriage, including vaccine-type pneumococci, has been underestimated in adults. Overall, our study highlights how improved understanding of the pneumococci circulating in the community setting can explain the continued pneumococcal disease due to vaccine serotypes among adults and supports the direct vaccination of older adults.

## Supplementary Material

ofaf008_Supplementary_Data

## Supplementary Data

Supplementary materials are available at *Open Forum Infectious Diseases* online. Consisting of data provided by the authors to benefit the reader, the posted materials are not copyedited and are the sole responsibility of the authors, so questions or comments should be addressed to the corresponding author.
