# Supplementary figures and images for: Detection of Pneumococcal Carriage in Asymptomatic Healthcare Workers
Source: Open Forum Infect Dis. 2025 Jan 15;12(2):ofaf008. doi: 10.1093/ofid/ofaf008 (PMC11800483; doi:10.1093/ofid/ofaf008)

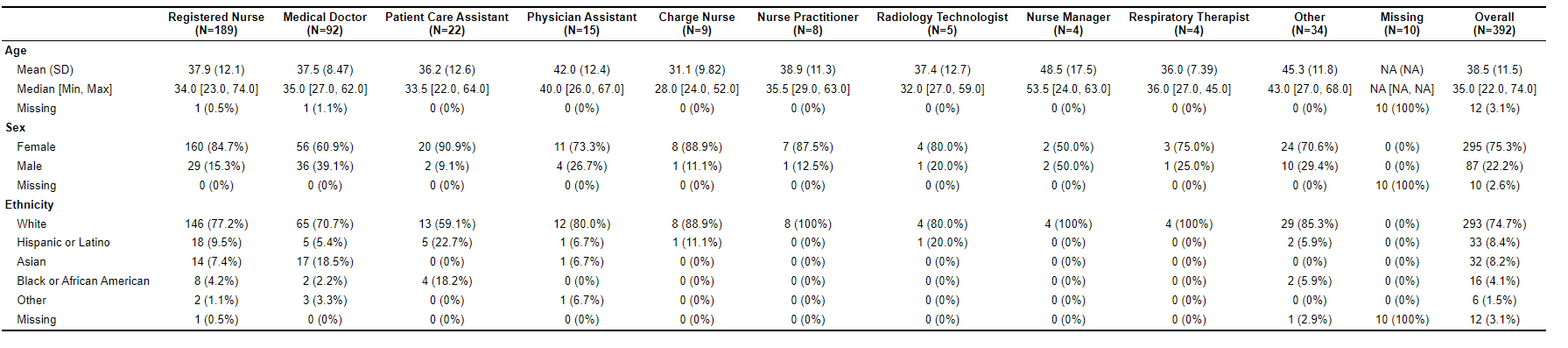

Supplement: ofaf008_Supplementary_Data [file ofaf008_supplementary_data.zip › SuppTable.png]
